# The contribution of beneficial wheat seed fungal communities beyond disease‐causing fungi: Advancing heritable mycobiome‐based plant breeding

**DOI:** 10.1111/1758-2229.70004

**Published:** 2024-11-11

**Authors:** Lindsey E. Becker, Marc A. Cubeta

**Affiliations:** ^1^ Department of Entomology and Plant Pathology, Center for Integrated Fungal Research North Carolina State University Raleigh North Carolina USA

## Abstract

Wheat (*Triticum* sp.) is a staple cereal crop, providing nearly a fifth of the world's protein and available calories. While fungi associated with wheat plants have been known for centuries, attention to fungi associated with wheat seeds has increased over the last hundred years. Initially, research focused on fungal taxa that cause seed‐borne diseases. Seeds act as a physical link between generations and host specialized fungal communities that affect seed dormancy, germination, quality, and disease susceptibility. Interest in beneficial, non‐disease‐causing fungal taxa associated with seeds has grown since the discovery of *Epichloë* in fescue, leading to a search for beneficial fungal endophytes in cereal grains. Recent studies of the wheat seed mycobiome have shown that disease, seed development, and temporal variation significantly influence the composition and structure of these fungal communities. This research, primarily descriptive, aims to better understand the wheat seed mycobiome's function in relation to the plant host. A deeper understanding of the wheat seed mycobiome's functionality may offer potential for microbiome‐assisted breeding.

## Graphical abstract

Seeds serve as a conduit between generations, hosting specialized fungal communities influenced by the maternal plant and the environment. Understanding the functions of the wheat seed mycobiome could facilitate microbiome‐assisted breeding of climate‐resilient wheat varieties.

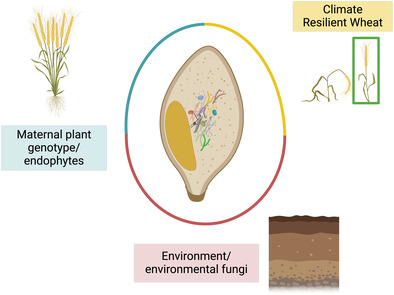

## INTRODUCTION

Wheat (*Triticum* spp.) represents a critical component of the world's food supply, with global production averaging 760 million metric tons over the past 10 years (USDA FAS—Production—Wheat). In the US farmers produced over 49 metric tons and harvested 14 million hectares in 2021 (Shiferaw et al., 2013; USDA ERS—Wheat Sector at a Glance). However, planted acreage in the United States has dropped precipitously over the past few decades (Vocke & Ali, 2013). In addition, drastic changes in precipitation rank high among farmer's concerns as the severity and frequency of extreme weather events heavily impact agricultural yields (Obembe et al., 2021; Zhao et al., 2022). Current models predict that as mean growing season temperatures increase, wheat production on a global scale is predicted to fall by 6% for every single degree centigrade increase (Asseng et al., 2015). Furthermore, under high greenhouse gas emission scenarios, heat stress is expected to occur in tandem with drought stress, which together are predicted to decrease wheat production in Southeast Asia and Africa by 15% (Pequeno et al., 2021). Liu et al. (2022) highlighted regional variation of wheat production within China and noted that modelling higher temperatures and lower precipitation resulted in greater uncertainty for modelling wheat yields. Sowing healthy seeds with high germination rates is critical for a crop with thin profit margins due to over‐estimated yields and increasing input costs in other areas such as machinery (Hiddink et al., 2023; Vocke & Ali, 2013).

**FIGURE 1:**
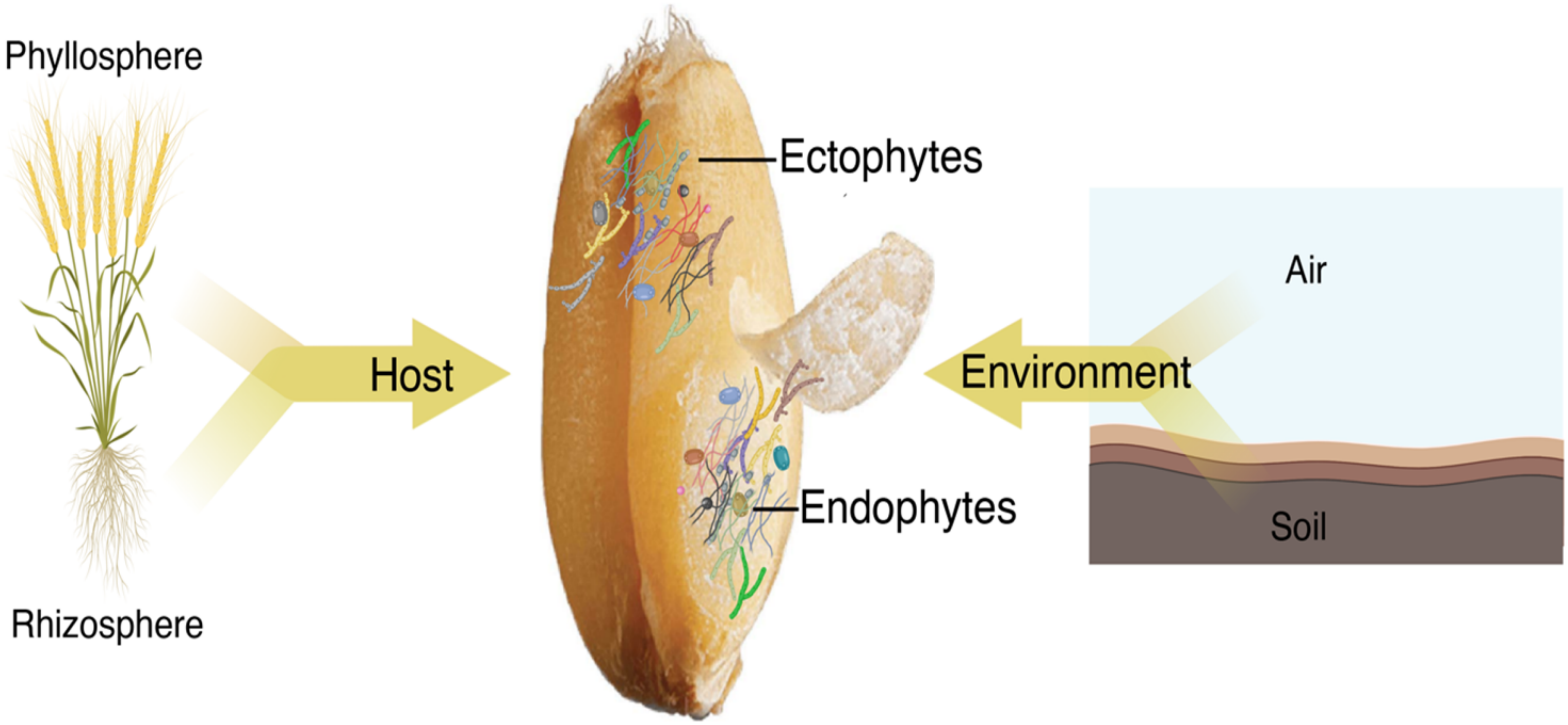
Schematic representation of factors influencing the composition and structure of the wheat seed mycobiome. Phyllosphere and Rhizosphere indicate mycobiomes associated with the maternal plant that can potentially be transmitted to the developing seed microbiome. Air and soil represent environmental sources of fungi that can also be introduced to the wheat seed through floral pathways or other means of introduction. Created with BioRender.com.

Plants harbour complex microbial communities composed of bacteria, fungi, archaea, and viruses that can potentially impact plant health in a positive, neutral, or negative fashion. Fungal communities (mycobiomes) residing in plants represent an inherent and overlooked resource to lessen crop losses and improve crop resilience to environmental change (Hussain et al., 2018; Trivedi et al., 2021). Plants harbour unique fungal communities within different tissues, with below and above‐ground tissues exhibiting differences in terms of composition and diversity (Gdanetz et al., 2021; Latz et al., 2021). During reproduction in a flowering plant, fungi often attempt to colonize the developing seed niche from other plant organs and the surrounding environment over time (Hertz et al., 2016; Nelson, 2018; Shade et al., 2017) (Figure 1). From a plant pathology perspective, crop production requires consistent monitoring of seed‐borne diseases that cause low germination in fields, produce harmful mycotoxins, grain spoilage, and potentially reduce international trade (Choudhury et al., 2017; Figueroa et al., 2018; Munkvold, 2009; Tuite & Foster, 1979). From an ecological perspective, seeds provide a specialized microhabitat that supports a unique community of fungi, acting to connect and promote the survival (perennation) of these organisms between plant generations. In this review, we focus on seed‐associated fungi since seed‐associated bacteria have been extensively covered in previous comprehensive reviews (Abdelfattah et al., 2023; Chen et al., 2022; Frank et al., 2017). Specifically, this review highlights the fungi that live within wheat seed often referred to as fungal endophytes (Petrini, 1991; Schardl et al., 2004). We acknowledge that this categorization is superficial as fungi can move both to and from the interior seed environment to the exterior seed environment. However, for clarity, we define vertical transmission in this review as the colonization of seeds by fungi from the endophytic tissue of the maternal plant, and horizontal transmission as the colonization of seeds by fungi from the surrounding external environment (Abdelfattah et al., 2023; Shade et al., 2017). This classification is useful for our purposes of describing the intimate association of fungi with seeds.

Seed‐associated fungi colonize developing plant tissues and are thought to influence plant microbiome assembly (Johnston‐Monje et al., 2021). Specifically, the primary symbiont hypothesis postulates that very few microbes are metabolically active within the seed based on low isolation rates in culture‐dependent studies. Therefore, microbes that are metabolically active play important functional roles in the developing plant (Newcombe et al., 2018). This hypothesis is further supported by a recent meta‐analysis of seed microbiome studies which revealed the seed mycobiome is often dominated by two or three fungal taxa in over 40 plant species (Simonin et al., 2022). However, there may be a benefit for seed‐associated fungi to exist in a dormant state within the seed and become active when the seed exits dormancy and germinates within a nutrient‐rich environment (Nelson, 2004). Microbial dormancy is the basis for Shade and Stopnisek's postulate that the presence of taxa within the seed is as important as abundance (Shade & Stopnisek, 2019). Additionally, the high abundance of fungal taxa within the seed is not always an indication of survival and successful transmission to the adult plant (Abdelfattah et al., 2021; Johnston‐Monje et al., 2021; Latz et al., 2021). The study of fungal transmission from seeds to developing plants in complex environments is largely complicated by reliance on the co‐occurrence of fungal taxa in seeds and seedlings, compared to more rigorously developed species‐specific primers (Johnston‐Monje et al., 2021; Latz et al., 2021; Mitter et al., 2017; Thapa et al., 2020). A better understanding of seed mycobiome composition, functionality, heritability, and transmission can improve our knowledge of how the seed mycobiome relates to the larger plant microbiome (Berg & Raaijmakers, 2018). Understanding the role that transmission plays in shaping the seed mycobiome will provide insight into how the seed microbial communities can be better deployed to aid plant host adaptation to rapidly changing environmental conditions (Trivedi et al., 2021).

Several fungal isolation studies in wheat have indicated that fungal communities of above‐ground wheat tissues, including leaves, stems, glumes, bracts, and kernels, differ greatly in terms of composition and structure from roots (Gdanetz & Trail, 2017; Larran et al., 2007; Sieber et al., 1988). Culture‐dependent studies provided evidence for developmental stages influencing fungal taxa isolated from wheat seeds at flowering and mealy ripe stages (Comby et al., 2016; Larran et al., 2007). Metabarcoding‐based studies, which are capable of detecting both culturable and nonculturable microbes, have corroborated that wheat aerial tissues represent a unique environment within the wheat plant, with seeds harbouring distinct fungal taxa (Gouka et al., 2022; Hertz et al., 2016; Latz et al., 2021).

Understanding factors that drive genetic diversity in the seed mycobiome is crucial for leveraging these fungal communities for crop improvement. While Comby et al. (2016) and Bakker and McCormick (2019) found that wheat genotypes impose limited influence on the wheat seed mycobiome, other studies highlight roles that plant genotypes can play in transmission efficiency. Abdelfattah et al. (2023) highlight two key phases that determine microbiome heritability in the seed; transmission of microbes from the maternal plant to the seed, and from the seed to the seedling. Gagic et al. (2018) demonstrated that seed‐borne transmission fidelity of *Epichloë* in ryegrass is predominately determined by plant host genotype, indicating that genetic factors can influence vertical transmission of fungal taxa. Current research investigating the heritability of plant‐associated microbiomes emphasizes the role of plant genetics (Escudero‐Martinez et al., 2022), root exudates (Sasse et al., 2018), and phenotypes (Wagner, 2021) in attracting beneficial microbes from the surrounding environment. However, less attention is given to understanding the relative impact of microbial communities intimately associated with seed and the developing plant. Previous seed microbiome studies of pumpkins (Adam et al., 2018), green foxtail (*Setaria viridis*) (Escobar Rodríguez et al., 2019), oilseed rape (Rybakova et al., 2017), and *Miscanthus* (Cope‐Selby et al., 2017) hinted at the implications of microbiome assisted breeding to select for beneficial members of the seed microbiome. Recently, Michl et al. (2024) examined the impact of breeding cycle, field site, and year on the seed microbiome of intermediate wheatgrass, noting that the community composition of seeds differed by breeding cycle in addition to field site and harvest year. These studies indicate the complexity and challenges associated with microbiome‐assisted breeding for enriching the seed microbiome.

## SEED‐ASSOCIATED FUNGI IN WHEAT

Fungi capable of producing mycotoxins, causing grain spoilage, and serving as causal agents of seed‐borne diseases have been well‐documented in wheat for centuries (Barnes et al., 2020; Wellings, 2011). Well‐known examples of seed‐borne fungal wheat diseases include loose smut, black point, blast, and Fusarium head blight (FHB) (Bockus et al., 2010). *Epichloë* isolates discovered within various members of Poaceae have sparked a search for similar seed endophytes of wild ancestors of wheat. While *Epichloë* species are known for mutualistic relationships with grasses, these fungi are also capable of producing toxic alkaloids resulting in reduced herbivory, causing ‘choke disease’ which reduces grass inflorescence development, reduces seed germination under water stress, and inducing a dwarfed phenotype in host plants during certain endophytic growth stages (Cheplick & Faeth, 2009; Gundel et al., 2006; Simpson et al., 2014; Zabalgogeazcoa et al., 2008). This complexity of fungal plant‐host interactions highlights the dual role of fungi as both beneficial and potentially harmful depending on environmental conditions and plant‐host interactions.

Research has revealed that some fungal seed endophytes present in wild ancestors of wheat are not maintained in domesticated wheat grown commercially today, suggesting that domestication of wheat has altered the seed microbiome (Marshall et al., 1999; Ofek‐Lalzar et al., 2016). A recent study assessed the fungal diversity of seeds from domesticated and wild wheat species using culture‐independent methods and found relatively low taxonomic diversity overall with little variation among species (Özkurt et al., 2020). In contrast, Marshall et al. (1999) used culture‐based approaches and noted differences, while Ofek‐Lalzar et al. (2016) employed both culture‐dependent and culture‐independent methods, observing differences in fungal communities between wild and domesticated wheat. These findings highlight the variability in results based on methodological approaches and underscore the importance of considering multiple methods when assessing fungal diversity.

Studies often report that two or three fungal taxa are most commonly isolated or detected within the seed, with *Cladosporium* and *Alternaria* occurring most commonly across 28 plant species, including wheat (Newcombe et al., 2018; Simonin et al., 2022). With regards to wheat, these dominant taxa vary slightly based on disease pressure, environment, and geography (Bakker & McCormick, 2019; Comby et al., 2016; Larran et al., 2007; Latz et al., 2021; Rojas et al., 2020). However, the majority of research studies report *Alternaria*, *Cladosporium*, *Epicoccum*, and *Parastagonospora* as dominant taxa of the wheat seed mycobiome, often representing 50% or higher relative abundance of reads within a seed sample (Larran et al., 2007; Rojas et al., 2020; Sieber et al., 1988; Simonin et al., 2022) (Table 1). Less abundant taxa include *Ascochyta*, *Aureobasidium, Blumeria*, *Fusarium*, and *Microdochium*, as well as basidiomycete yeast‐forming fungi including *Filobasidium*, *Sporobolomyces*, and *Vishniacozyma* (Gouka et al., 2022; Larran et al., 2007; Sieber et al., 1988). The majority of fungi in the Basidiomycota known to associate with wheat seeds are thought to exist in a yeast‐like form. A recent meta‐analysis conducted by Gouka et al. (2022) noted yeast and yeast‐like taxa associated with wheat seeds, with some taxa exhibiting antagonistic activity against plant pathogens in culture. These yeasts or yeast‐like taxa included *Aureobasidium*, *Bullera*, *Filobasidium*, *Naganishia*, *Saccharomyces*, and *Sporobolomyces*. We consider all filamentous and yeast fungal taxa associated with wheat seed to be of interest, especially those reported in multiple cultivars, vertically transmitted, span geographic locations, and associated with abiotic and biotic stress conditions. We note that not all seed‐associated fungi will not exhibit mutualist or pathogenic traits, but rather are well‐adapted to the seed niche and exist in a commensal state.

**TABLE 1:** A literature search of studies examining fungal endophytes identified in wheat seeds (*Triticum* spp.) was conducted. Fungal genera are listed, with their corresponding phylum and class level taxonomy. Identification methods used to capture fungal taxa are indicated as culture dependent (Dep.) or culture independent (Ind.). References list the studies in which each fungal genus is reported.

Fungal genera	Phylum	Class	Approach	References
*Alternaria*	Ascomycota	Dothideomycetes	Both	Ofek‐Lalzar et al. (2016), Larran et al. (2007), Sieber et al. (1988), Comby et al. (2016), Latz et al. (2021), Bakker and McCormick (2019), Rojas et al. (2020)
*Aureobasidium*	Ascomycota	Dothideomycetes	Both	Ofek‐Lalzar et al. (2016), Comby et al. (2016), Rojas et al. (2020)
*Cladosporium*	Ascomycota	Dothideomycetes	Both	Ofek‐Lalzar et al. (2016), Larran et al. (2007), Sieber et al. (1988), Comby et al. (2016), Latz et al. (2021), Bakker and McCormick (2019), Rojas et al. (2020)
*Stemphylium*	Ascomycota	Dothideomycetes	Both	Ofek‐Lalzar et al. (2016), Larran et al. (2007)
*Fusarium*	Ascomycota	Sordariomycetes	Both	Ofek‐Lalzar et al. (2016), Larran et al. (2007), Sieber et al. (1988), Comby et al. (2016), Latz et al. (2021), Bakker and McCormick (2019), Rojas et al. (2020)
*Arthrinium*	Ascomycota	Sordariomycetes	Dep.	Larran et al., 2007
*Bipolaris*	Ascomycota	Dothideomycetes	Dep.	Larran et al. (2007), Bakker and McCormick (2019)
*Candida*	Ascomycota	Saccharomycetes	Dep.	Larran et al. (2007)
*Chaetomium*	Ascomycota	Sordariomycetes	Dep.	Larran et al. (2007)
*Cochliobolus*	Ascomycota	Dothideomycetes	Dep.	Larran et al. (2007)
*Epicoccum*	Ascomycota	Dothideomycetes	Both	Larran et al. (2007), Sieber et al. (1988), Comby et al. (2016), Latz et al., 2021, Bakker and McCormick (2019)
Parastagonospora	Ascomycota	Dothideomycetes	Both	Sieber et al. (1988), Rojas et al. (2020)
*Idriella*	Ascomycota	Leotiomycetes	Dep.	Sieber et al. (1988)
*Rhizoctonia*	Basidiomycota	Agaricomycetes	Dep.	Sieber et al. (1988)
*Didymella*	Ascomycota	Dothideomycetes	Dep.	Sieber et al. (1988)
*Trametes*	Basidiomycota	Agaricomycetes	Dep.	Comby et al. (2016)
*Rhodosporidium*	Basidiomycota	Exobasidiomycetes	Dep.	Comby et al. (2016)
*Drechslera*	Ascomycota	Dothideomycetes	Dep.	Comby et al. (2016)
*Diaporthe*	Ascomycota	Sordariomycetes	Dep.	Comby et al. (2016)
*Acremonium*	Ascomycota	Sordariomycetes	Ind.	Latz et al. (2021)
*Monographella*	Ascomycota	Sordariomycetes	Ind.	Latz et al. (2021)
*Mycosphaerella*	Ascomycota	Dothideomycetes	Ind.	Latz et al. (2021), Bakker and McCormick (2019)
*Penicillium*	Ascomycota	Eurotiomycetes	Both	Latz et al. (2021), Agoussar et al., (2021)
*Malassezia*	Basidiomycota	Malasseziomycetes	Ind.	Bakker and McCormick (2019)
*Filobasidium*	Basidiomycota	Tremellomycetes	Ind.	Bakker and McCormick (2019)
*Holtermanniella*	Basidiomycota	Tremellomycetes	Ind.	Rojas et al. (2020)
*Itersonilia*	Basidiomycota	Tremellomycetes	Ind.	Rojas et al. (2020)
*Vishniacozyma*	Basidiomycota	Tremellomycetes	Ind.	Rojas et al., 2020
*Sporoboloymyces*	Basidiomycota	Microbotryomycetes	Ind.	Rojas et al. (2020)
*Puccinia*	Basidiomycota	Pucciniomycetes	Ind.	Rojas et al. (2020)
*Leucosporidium*	Basidiomycota	Microbotryomycetes	Ind.	Rojas et al. (2020)
*Botrytis*	Ascomycota	Leotiomycetes	Ind.	Rojas et al. (2020)

### Factors that influence the wheat seed mycobiome

Understanding the factors that shape the wheat seed mycobiome is crucial for leveraging microbial communities to improve crop productivity and resilience. Abiotic and biotic factors play substantial roles in influencing the composition and structure of seed fungal communities.

## TEMPORAL AND DEVELOPMENTAL STAGE

Temporal variation has been reported to contribute to seed mycobiome composition. Bakker and McCormick (2019) found that year was a significant driver of variation in wheat seed fungal community structure compared to FHB disease incidence. However, the percentage of variance explained by each dependent variable was not reported. Hertz et al. (2016) demonstrated the impact and role that the wheat seed developmental stage plays in determining seed mycobiome composition and structure. Their research highlighted the dominance of the basidiomycete yeast *Sporobolomyces* in early seed development, with the Ascomycete *Alternaria* dominating the seed mycobiome at the ripening stage. These findings were corroborated by Comby et al. (2016), who examined seeds, brachs, glumes, leaves, and roots of two wheat cultivars and recorded the structuring of plant microbiome by developmental stages, with heading and flowering separating from ripening stages. In addition, Rojas et al. (2020) also noted changes in species richness among tissues when comparing pre‐anthesis to ripening stages, which is consistent with the physiological changes occurring within the seed as the plant senesces (Nelson, 2018).

## ABIOTIC FACTORS

When investigating abiotic factors that shape the wheat seed mycobiome, Latz et al. (2021) reported that wheat plants grown in a greenhouse exhibited a drastically different fungal community compared to plants grown in a field adjacent to the greenhouse. Their observations suggest the environmental plasticity of wheat seeds, with slight environmental variability between indoor and outdoor conditions contributing to a reduction of species richness and a significant shift in community structure. A recent study by Azarbad et al. (2022) examined the impacts on the wheat seed mycobiome of wheat grown in soil with contrasting drought stress histories, revealing a stable and robust mycobiome despite varying environmental conditions.

## BIOTIC FACTORS

When examining biotic factors that shape the wheat seed mycobiome, Bakker and McCormick (2019) and Rojas et al. (2020) conducted field‐based wheat inoculations with the causal agents of FHB, a devastating fungal pathogen that infects flowers and colonizes developing seeds (Bai & Shaner, 2004). FHB causal agents produce mycotoxins within the grain, consequently reducing grain quality and overall yield. Rojas et al. (2020) observed that control and Fusarium‐infected symptomless grains exhibited similar fungal community structures, while Fusarium‐infected symptomatic grains differed in fungal community structure and exhibited decreased species richness. Bakker and McCormick (2019) conducted a single‐seed experimental investigation to associate FHB and mycotoxin concentrations with impacts on the wheat seed fungal community and reported that fungal diversity was negatively correlated with increased Fusarium abundance in the seed. Similarly, Latz et al. (2021) and Comby et al. (2016) found that wheat pedigree and disease susceptibility phenotypes of wheat did not perturb the mycobiome composition or structure, respectively.

## CONFLUENCE OF STRESSORS

In addition to examining other abiotic and biotic factors, an examination of the confluence of stressors synergistically is critical, as drought often occurs in tandem with elevated air temperature stress, and disease pressure is often increased in high relative humidity environments (Chaloner et al., 2021; Garrett et al., 2006; Savary et al., 2017; Tang et al., 2017; Zhao et al., 2022). Unexplored aspects of wheat seed mycobiomes in the quest to study vertical and horizontal transmission may require seed‐to‐seed multi‐generational experimental designs, as conducted in lentils and tomatoes (Bergna et al., 2018; Morales Moreira et al., 2021). Multi‐generational experiments may also provide much‐needed insight into the resilience of the seed mycobiome following abiotic or biotic stress (Becker & Cubeta, 2024; Shade, 2023). Gaining insight into the role that geography and environment play in shaping the seed mycobiome will also be useful for determining the generalizability of results and breadth of applicability (Schloss, 2018). As wheat seeds are generally found to have similar fungal taxa and community structure across several continents, results could be broadly applied beyond the small geographical region where studies were conducted.

## REPRODUCIBILITY AND REPLICATION

The reviewed literature highlights the need for replicated experiments conducted over multiple years in the greenhouse and field to examine the stability of responses, as exemplified by Bakker and McCormick (2019). Gdanetz et al. (2021; 2017) emphasized the importance of including robustly replicated plots when examining crop‐associated microbiomes of wheat, corn, and soybean. Consistent replication is key, as evidenced in the controlled environment experiments by Latz et al. (2021), even though environmental stochasticity cannot be easily controlled. Knight et al. (2018) and Schloss (2018) have cautioned researchers to pay careful attention to reproducibility, which can be addressed by depositing sequence data in universally accessible databases coupled with accompanying metadata and by utilizing amplicon sequence variants (ASV) that report the sequence rather than an ambiguous operational taxonomic unit (Callahan et al., 2016). Standard best practices, such as reporting detailed sample preparation, DNA extraction approaches, negative controls, and sequencing methods, are critical for reproducibility and meta‐analyses that provide increased insight into plant mycobiome trends across multiple lines of investigation (Simonin et al., 2022).

In summary, our current knowledge of the wheat seed mycobiome indicates that developmental stage, disease pressure, experimental conditions, and field season are key factors that likely influence mycobiome composition and structure. Further research is needed to explore these factors in more detail and to develop strategies for leveraging the wheat seed mycobiome to enhance crop productivity and resilience.

## TRANSMISSION OF SEED‐ASSOCIATED FUNGI TO THE DEVELOPING PLANT

From a bacterial perspective, a small but significant cohort of seed‐associated bacteria persist in early wheat seedling stages (Johnston‐Monje et al., 2021; Walsh et al., 2021). Inoculation of wheat flowers with a beneficial bacteria known to promote higher yield resulted in successful inoculation of seeds and detection in roots and shoots of the next generation (Mitter et al., 2017). However, less is known about how beneficial fungal endophytes of wheat seeds are maintained at the seedling stage and within the developing plant (Özkurt et al., 2020). There is a long history of research on seed‐transmitted fungal pathogens of wheat, including fungi that cause FHB, septoria nodorum blotch, loose smut, and other seed‐borne diseases that indicate the persistence of seed‐associated fungi in the developing wheat seedling (Bockus et al., 2010; Majumder et al., 2013). Furthermore, many of the fungal taxa identified in seeds are closely related to disease‐causing species that infect wheat. It is imperative to conduct virulence testing using Koch's postulates, to establish the disease‐causing activity of individual fungal isolates detected in wheat seeds and avoid assumptions based solely on taxonomic classification.

Recent studies have advanced our understanding of fungal endophyte transmission. Sharon et al. (2023) investigated the transmission mode and assembly of seed fungal endophyte communities in wheat and its wild relatives. Their research utilized fungal amplicon metabarcoding to confirm the vertical transmission of fungal taxa from seed to seed but also highlighted the surrounding environment as a primary source for members of the seed microbiome. Additionally, Matušinsky et al. (2024) developed a qPCR‐based diagnostic assay using species‐specific primers to track colonization dynamics and distribution of the endophytic fungus *Microdochium bolleyi* in wheat and purple false brome grass *Brachypodium distachyon*. Their research demonstrated that *M. bolleyi* colonizes the roots and basal parts of the stem but does not colonize the leaves or ears and is not transmitted to the next generation via seeds. This study underscores the challenges in real‐time monitoring and tracking endophyte presence within host plants and highlights the importance of developing species‐specific fungal primers for precise evaluation.

Currently, in microbiome research there is an examination of co‐occurrence at the ASV level for developing conclusions about microbial sources and microbial transmission over time (Hertz et al., 2016; Latz et al., 2021). To better establish transmission of fungal endophytes from seed to seedling it may be prudent to borrow tactics from diagnostic plant pathology and design species‐specific fungal primers (Thapa et al., 2020). This approach has been applied by Mitter et al. (2017) who used species‐specific bacterial primers to examine transmission of an inoculated bacteria in the seed, roots, and leaves of progeny plants. Transformation methods, such as the use of green fluorescent protein, m‐cherry, and enhanced cyan‐fluorescent protein labelling, enable tracking of the development of mycelium in plant tissues and provide a powerful tool for visualizing and studying fungal endophyte colonization and movement within the host (Liu et al., 2018; Zhu et al., 2022). Moreover, the development of CRISPR‐based diagnostic assays offers the potential for identifying endophytes of interest in complex *in planta* samples, allowing for early detection and monitoring prior to disease symptom development (Foster et al., 2024). While these methods present robust approaches, they may not always be feasible due to constraints associated with working with non‐culturable fungi and lack of genomic resources. Therefore, integrating multiple methodologies, including genomic imaging and surveillance techniques, will provide a more comprehensive understanding of fungal endophyte transmission and dynamics within wheat tissues in a spatial and temporal framework.

## CONCLUDING REMARKS

In the European Union, limitations on fungicide use for seed coat treatment are already in place (Lamichhane et al., 2020). If similar legislation is enacted in the United States, a valuable disease management strategy will be mitigated or eliminated. To address grower needs, combat early seedling diseases, and abiotic stress, and decrease fertilizer use, exploring seed‐associated endophytic fungi as an alternative or complementary strategy is warranted. Current studies are examining fungal endophytes sourced from seeds to combat diseases such as FHB (Noel et al., 2022). Most biological seed treatments focus on external applications to the external seed coat, which can improve germination and provide protection against early‐season seedling diseases (Schiltz et al., 2015). However, it can be challenging for an externally introduced organism to establish, function, and survive within the rhizosphere and surrounding soil (Mitter et al., 2019).

A major impediment to the deployment of fungal microbial communities (rather than a single organism) is ensuring that the introduced microbe is well‐suited and competitive within the environment (Albright et al., 2022). Seed microbiome engineering, either by microbiome‐assisted engineering (Adam et al., 2018; Berg & Raaijmakers, 2018), or inoculation of seeds via flowers (Mitter et al., 2017), provides a potential solution to the aforementioned limitations of agricultural microbiome applications by allowing the beneficial fungi to persist and provide long‐term benefits for the plant host. These approaches face many challenges such as imperfect transmission of fungal endophytes via floral pathways and variable transmission of seed‐associated fungi across generations. Future studies may utilize seed traits for genome‐wide association studies and quantitative trait loci studies to examine the correlation of microbial abundance with traits and general heritability of the seed microbiome, as are currently studied in the context of the rhizosphere microbiome (Deng et al., 2021; Escudero‐Martinez et al., 2022; Wang et al., 2022). We postulate that targeted isolation of fungi of interest from seeds could lead to the identification of beneficial fungi well suited to the seed niche environment, and therefore prime candidates for seed microbiome engineering. Such efforts have a multitude of benefits, as beneficial fungi introduced as endophytes may have the potential to co‐exist with current seed treatments, but may also prove fruitful for providing growers with solutions if protective and systemic chemical seed coat treatments are not an option.

## AUTHOR CONTRIBUTIONS

**Lindsey E. Becker:** Conceptualization; writing – original draft; writing – review and editing; funding acquisition. **Marc A. Cubeta:** Writing – review and editing; funding acquisition; supervision.

## CONFLICT OF INTEREST STATEMENT

The authors declare no conflicts of interest.

## Data Availability

Data sharing not applicable to this article as no datasets were generated or analysed during the current study.
